# Assessment of Risk of Rheumatoid Arthritis Among Underground Hard Rock and Other Mining Industry Workers in Colorado, New Mexico, and Utah

**DOI:** 10.1001/jamanetworkopen.2022.36738

**Published:** 2022-10-17

**Authors:** Paul D. Blanc, Laura Trupin, Edward H. Yelin, Gabriela Schmajuk

**Affiliations:** 1 Department of Medicine, University of California, San Francisco; 2 Department of Medicine, San Francisco Veterans Affairs Health Care System, San Francisco, California

## Abstract

**Importance:**

Respirable silica exposure has been strongly and consistently linked to rheumatoid arthritis (RA) among foundry workers, persons in the construction trades, stone crushers and drillers, and coal miners. However, risk of RA in hard rock mining has not been thoroughly investigated.

**Objective:**

To analyze occupational risk of RA in hard rock miners in Colorado, New Mexico, and Utah.

**Design, Setting, and Participants:**

This cross-sectional survey study estimated the association between mining industry work and reported RA in a random-digit telephone survey of men 50 years or older living in selected counties with elevated levels of pneumoconiosis mortality (N = 1988). The survey was conducted between January 12 and May 4, 2021.

**Exposures:**

Underground hard rock and other mining and related mineral-processing occupations.

**Main Outcomes and Measures:**

Report of a clinician diagnosis of RA further defined by treatment with corticosteroids or disease-modifying antirheumatic drugs. Risk was estimated using logistic regression.

**Results:**

The analytic sample of 1988 men (survey response rate, 11.1% of all contacts) had a mean (SD) age of 68.6 (10.1) years. Underground hard rock mining was reported by 118 (5.9%); underground mining of other types, predominantly coal mining (no concomitant hard rock), 62 (3.1%); and surface mining or ore processing (no underground), 262 (13.2%). Adjusting for age and smoking and accounting for nonmining silica exposure, mining employment was associated with increased odds of corticosteroid-treated RA (n = 89) (odds ratio, 4.12 [95%, 2.49-6.81]). The odds were similar for RA treated with disease-modifying antirheumatic drugs (n = 80) (odds ratio, 3.30 [95% CI, 1.93-5.66]).

**Conclusions and Relevance:**

In this cross-sectional survey study, workers in hard rock and other underground mining and surface mining occupations experienced 3- to 4-fold increased odds of RA. These findings suggest that clinicians should consider patients with relevant work exposures as at higher risk for developing RA.

## Key Points

**Question:**

Is hard rock underground mining and other mining employment associated with a risk of rheumatoid arthritis (RA)?

**Findings:**

In this cross-sectional population survey study that included 1988 mining industry workers, 442 workers with mining exposure experienced 3- to 4-fold increased odds of RA.

**Meaning:**

These findings suggest that mining industry work is associated with RA and that clinicians should consider occupational history when assessing this condition.

## Teaser

This cross-sectional survey study assesses whether hard rock underground mining and other mining industry occupations are associated with increased risk of rheumatoid arthritis using a population-based survey of men in counties of Colorado, New Mexico, and Utah with mining activity and high rates of silicosis-related mortality.

## Introduction

Respirable silica exposure has been strongly and consistently associated with rheumatoid arthritis (RA) across a variety of occupations.^1,2^ Examples of at-risk jobs include foundry work, construction trades, and stone crushing and drilling. We previously observed that underground coal miners from the Appalachian region of the Eastern US had 3-fold or greater odds of RA.^3,4^ This risk is presumably due to silica coexposure from underground coal extraction in which silica-laden dust from beyond the coal seam routinely contaminates the workers’ breathing zones.^5^ Coal mining, especially in Great Britain, has long been recognized as a risk factor for RA, often referred to as Caplan syndrome in that context.^6^

Beyond coal, underground metal and other hard rock mining also is an important source of silica exposurse.^7,8^ Despite this exposure, RA risk in hard rock mining has received scant attention by researchers, clinicians, and policy makers. A single 1995 mortality study of South Dakota gold miners^9^ identified an increased risk of overall arthritis-related mortality but did not consider RA specifically. A study of RA in South African gold miners was published in the 1980s,^10^ whereas a 1979 letter to the editor reported the prevalence of RA among silicotic metal miners from Quebec.^11^ These studies appear to constitute the entire published literature on hard rock mining risk for RA.

We hypothesized that hard rock underground mining would be associated with increased RA risk, along with other occupational sources of silica exposure, including coal mining. Our research objective was to study this question, performing a population-based survey of persons living in counties in Colorado, New Mexico, and Utah with mining activity and high rates of silicosis-related mortality.

## Methods

### Data Source

Data for this cross-sectional survey study were derived from a cross-sectional, random-digit dial population-based telephone survey conducted between January 12 and May 4, 2021. The random-digit dial survey was conducted by Davis Research and adhered to the guidelines of the American Association for Public Opinion Research (AAPOR). The full survey instrument is provided as an eAppendix in the Supplement. The study was approved by the Institutional Review Board of the University of California, San Francisco; all participants provided verbal consent to proceed with the interview.

**Figure 1.**
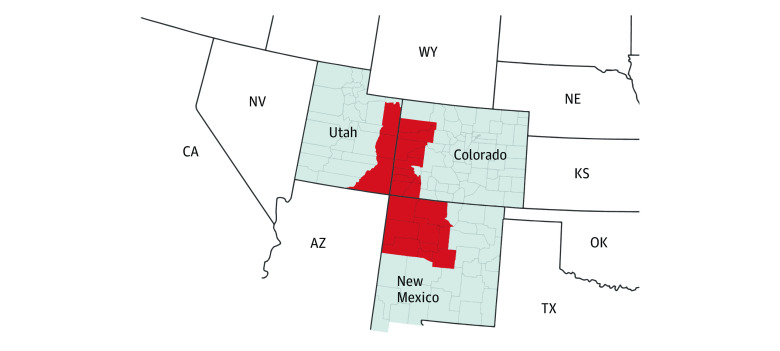
Sampled Counties in Colorado (n = 10), New Mexico (n = 11), and Utah (n = 3) Based on Elevated Pneumoconiosis-Related Mortality Red area of map indicates the sampled counties.

The random-digit dial sample included both landline and cellular telephones, targeting exchanges likely to be in counties in Utah, New Mexico, and Colorado with historically high rates of mortality due to silicosis. We identified the targeted areas using data from the National Institute for Occupational Safety and Health.^12^ Although not selected based on geographical contiguity, the 26 counties included in the study were adjacent to each other (Figure 1).

We limited eligibility for survey participation to men 50 years and older who spoke English or Spanish, had a history of or any current labor force participation, and confirmed current residence in one of the targeted counties. We based both the sampling methods and the survey interview on our previous research focusing on the risk of RA associated with coal mining in the Appalachian region of the US.^3,4^

### Study Sample

From 83 014 call attempts, we made 18 180 contacts with potential participants (eFigure in the Supplement). A total of 5841 individuals declined to participate, most before eligibility could be established. A further 10 413 were ineligible owing to age, sex, preferred language (not English or Spanish), lack of employment history, or current residence outside the catchment area. A total of 2000 responded to the survey (11.0% of contacts made; 25.7% of contacts after excluding the 10 413 known to be ineligible). Our target final number was based on our previous experience surveying coal miners in Appalachia.^3,4^ Race and ethnicity data by self-report were collected in the survey and were relevant to this mining population in particular because of past patterns of employment in mining.

### Survey Instrument

On average, study interviews did not exceed 18 minutes in aggregate average. Study interviews contained items addressing employment, smoking history, sociodemographic characteristics, and arthritis and related diagnoses. The survey ascertained duration and type of mining experience, along with exposure to inhaled dusts in non–coal mining jobs. The health sections queried whether the respondent had ever received a diagnosis of arthritis of any kind from a health care professional, with follow-up items specifying RA, psoriatic arthritis, or gout. The survey also elicited diagnoses of other autoimmune conditions, including systemic lupus erythematosus, psoriatic arthritis, and systemic sclerosis. Interviewers asked about joint swelling, stiffness, or pain, regardless of reported diagnoses. Finally, the survey included a series of questions about immunosuppressive medications used to treat arthritis.

### Exposure Classification

We defined mining exposure based on survey-reported occupational history. We asked about any underground hard rock mining employment, with a follow-up list eliciting the specific minerals mined. Respondents who did not endorse any of these (silver, gold, copper, molybdenum, zircon, or uranium) had an open-ended option to identify their hard rock mining exposure. Other mining activities named in these responses that were likely to involve silica exposure were considered hard rock exposures. Those reporting underground shale oil mining or gilsonite, however, were combined with underground coal mining, ascertained in a separate series of questionnaire items directed at underground (and surface) coal mining. Those who identified other mining activities with negligible silica exposure (ie, trona) and those who reported no specific exposure were not classified as exposed.

Additional survey items inquired about open-pit mining, ore processing, and quarry work. Other silica dust exposure (not from coal mining) was defined by affirmative responses to any of a list of specific job tasks, including work with silica, sand, or concrete dust; sandblasting; foundry work; concrete finishing, cutting, or drilling; or masonry work or tuckpointing. For each type of exposure, the survey included a follow-up question about the number of years spent doing that work. We did not elicit global occupational histories and thus did not apply a job exposure matrix to generate an alternate exposure measure.

We created a hierarchical, mutually exclusive classification of silica exposure. Any specifically identified underground hard rock mining constituted the first category, taking precedence over all other reported sources of silica exposure. Any underground soft rock mining constituted the next category and included coal, shale oil, and gilsonite, followed by any surface mining, ore refining, or quarrying. Silica exposure exclusively from nonmining sources constituted the final category. We also created an aggregate classification of any mining exposure and any nonmining exposure. For both exposure variables, those with all other types of current or past employment constituted the unexposed group.

### Disease Classification

We defined arthritis by an affirmative response to the primary stem question about receiving a diagnosis of arthritis. We defined RA based on a follow-up item about type of arthritis, restricted to those reporting having received oral or injected glucocorticoids for joint symptoms. We also created a more restricted definition based on prolonged use of glucocorticoids, defined as at least 3 times per week for at least 3 months’ duration. A more specific definition for RA required a diagnosis of RA and report of receiving at least 1 of a standard list of disease-modifying antirheumatic drugs (DMARDs), including conventional synthetic DMARDS (methotrexate, sulfasalazine, hydroxychloroquine sulfate, azathioprine, or leflunomide) or biologics or targeted small molecules approved for the treatment of RA (etanercept, adalimumab, infliximab, golimumab, certolizumab, tocilizumab, abatacept, rituximab, tofacitinib, upadacitinib, or baricitinib). We also created an alternative definition including those who reported RA and either glucocorticoid or DMARD use. We categorized non-RA arthritis by a positive response to the initial arthritis question without meeting the main study criteria for RA. This category is likely to be predominantly degenerative arthritis (osteoarthritis) but includes reported RA without use of glucocorticoids or DMARDs as well as infrequent reports of other autoimmune or crystalline arthritis.

### Smoking Classification

Cigarette smoking exposure assessment included age at start, number of years smoked, and median number of cigarette packs smoked per day. We categorized participants as current, former, or never smokers. Respondents who had quit smoking fewer than 3 years before the interview were considered recent smokers and were included in the same category as current smokers.

### Statistical Analysis

Using multivariable logistic regression analyses, we modeled the risk of RA (defined by use of glucocorticoids or DMARDs) and non-RA arthritis associated with underground hard rock mining, underground coal mining, surface mining, and other nonmining silica exposure compared with the unexposed group. The models adjusted for age, race and ethnicity (non-Hispanic White vs all others), and smoking status (current, former, or never). Respondents who did not answer the smoking status questions (n = 5) were excluded from these analyses. We also excluded respondents whose only reported underground exposure was trona, as well as those who did not report any specific work history that would allow for categorization as hard rock or coal and/or shale exposure (n = 7), resulting in a final analytic sample of 1988. There were no other key variables with missing data.

We tested the aggregate classification of any mining exposure to silica and nonmining silica exposure in a series of models in which the dependent variables were the multiple definitions of RA we applied (1) requiring glucocorticoid use, (2) requiring DMARD use, (3) requiring long-term glucocorticoid use, or (4) either glucocorticoid or DMARD use. Because there could have been clustering by geography, we reevaluated the main multivariable models using generalized estimating equations accounting for the state from which the counties were drawn.

To reduce the chance of misclassification bias, all models of RA (any definition) or non-RA arthritis excluded respondents who reported arthritis but did not meet the criteria for the diagnosis under consideration. For example, models of RA with glucocorticoid use excluded respondents who reported arthritis but not RA or who reported RA but not glucocorticoid use. Thus, the reference population in each model consisted only of individuals without arthritis by any definition. Statistical analyses were performed using SAS, version 9.4 (SAS Institute, Inc), and Stata, version 15 (StataCorp LLC).

## Results

We analyzed responses for 1988 survey participants, all of whom by study eligibility criteria were men 50 years or older (mean [SD] age. 68.6 [10.1] years) with a history of labor force participation. Participant recruitment and exclusions are shown in the eFigure in the Supplement. Table 1 presents sociodemographic characteristics and cigarette smoking status for the study cohort. Most respondents (1124 [56.5%]) resided in the counties sampled in Colorado, with the remainder in New Mexico (515 [25.9%]) and Utah (349 [17.5%]). Although most participants were non-Hispanic White (1643 [82.6%]), Hispanic individuals accounted for 208 respondents (10.5%), and American Indian or Alaska Native individuals accounted for 56 (2.8%). Just more than half (1006 [50.6%]) were never smokers. Underground hard rock mining was reported by 118 (5.9%); underground mining of other types, predominantly coal mining (no concomitant hard rock), 62 (3.1%); and surface mining or ore processing (no underground), 262 (13.2%).

**Table 1.** Respondent Characteristics

Characteristic	Respondent data (N = 1988)^a^
Sociodemographic	
Age, mean (SD), y	68.6 (10.1)
Race and ethnicity	
American Indian or Alaska Native	56 (2.8)
Asian	12 (0.6)
Hispanic	208 (10.5)
Non-Hispanic Black	13 (0.7)
Non-Hispanic White	1643 (82.6)
Other or unknown^b^	56 (2.8)
State of residence	
Colorado	1124 (56.5)
New Mexico	515 (25.9)
Utah	349 (17.5)
Currently employed	758 (38.1)
Cigarette smoking	
Never	1006 (50.6)
Former	780 (39.2)
Current and recent^c^	202 (10.2)
Packs/d, median (IQR)	1.0 (0.5-1.0)
Duration of smoking among ever smokers^d^	
≤20 y	495 (51.6)
>20 y	465 (48.4)
Pack-years, median (IQR)^e^	18 (6-40)
Sources of exposure (not mutually exclusive)	
Any underground hard rock mining exposure	118 (5.9)
Uranium or zircon mining	62 (3.1)
Any other metal mining	83 (4.2)
Duration of hard rock mining, median (IQR), y^f^	2 (1-10)
90th percentile, y	25
Any underground soft rock mining^g^	81 (4.1)
Duration of soft rock mining, median (IQR), y	6 (3-22)
90th percentile, y	38
Any surface mining, ore processing, refining	336 (16.9)
Any nonmining occupational silica exposure	573 (28.8)
Exposure source (nonmutually exclusive categories)^h^	
Silica exposure from mining sources	442 (22.2)
Any underground hard rock mining exposure	118 (5.9)
Underground soft rock (no hard rock)	62 (3.1)
Surface work (no underground)	262 (13.2)
Silica exposure from nonmining sources only	348 (17.5)
No mining or silica exposure	1198 (60.3)
Arthritis type and frequency	
No diagnosis of arthritis reported	1073 (54.0)
Any arthritis reported	915 (46.0)
Arthritis, exclusive of RA	683 (34.3)
Any RA reported	232 (11.7)
RA with DMARDs or corticosteroids^i^	118 (5.9)
RA with corticosteroids	89 (4.5)
RA with long-term corticosteroids^j^	49 (2.5)
RA with DMARDs^i^	80 (4.0)

Abbreviations: DMARD, disease modifying antirheumatic drug; RA, rheumatoid arthritis.

^a^ All respondents were men 50 years or older with a history of previous labor force participation. Unless indicated otherwise, data are expressed as No. (%) of respondents. Percentages have been rounded and may not total 100.

^b^ Includes multiple races or ethnicities and declined to state.

^c^ Includes those who stopped smoking in the past 3 years.

^d^ Missing for 22 respondents.

^e^ Missing for 63 respondents.

^f^ Missing for 1 respondent.

^g^ Includes coal, shale oil, and gilsonite.

^h^ Categories yield a sum greater than that for any exposure (n = 790).

^i^ See the Disease Classification subsection of the Methods section for included medications.

^j^ Indicates 3 or more times per week for 3 or more months.

Mining and other sources of silica exposure also are shown in Table 1. Altogether, more than 1 in 5 respondents (442 [22.2%]) reported underground or surface mining experience. Those with underground hard rock mining experience accounted for 118 of 442 with such exposures. The duration of hard rock mining employment was relatively brief (median, 2 [IQR, 1-10] years), whereas for soft rock mining the median was 6 (IQR, 3-22) years. Other occupational exposures likely to involve silica were nearly as common as the mining industry (348 [17.5%] of the study respondents). Table 1 further presents the frequencies of arthritis for differing definitions of disease. Eighty-nine respondents reported a diagnosis of RA from a clinician and treatment of joint pain with corticosteroids, whereas 80 reported RA and treatment with a DMARD. The 2 definitions overlapped; 51 respondents reported treatment with both corticosteroids and DMARDs, whereas 38 reported corticosteroids only and 29 reported DMARDs only.

The risks of arthritis by source of silica exposure are presented in Table 2. For RA defined by corticosteroid use, there was a greater than 3-fold increased odds of disease associated with underground hard rock mining (odds ratio [OR], 3.21 [95% CI, 1.45-7.10]), surface mining (OR, 3.74 [95% CI, 2.07-6.75]), and silica from other sources (OR, 3.40 [95% CI, 1.84-6.27]), whereas the odds associated with coal mining were increased greater than 9-fold (OR, 9.74 [95% CI, 3.89-24.42]). Using a definition of disease requiring DMARD treatment, the point estimate of the odds of RA was lower for underground mining (OR, 1.91 [95% CI, 0.71-5.12]) and was not significant. In these analyses, neither current nor past smoking was associated with RA. Arthritis other than RA was associated with underground mining other than hard rock mining, surface mining, and processing and other silica exposure jobs, but in all cases with lower point estimates of risk compared with RA (eg, OR for underground hard rock mining, 1.38 [95%, 0.93-2.05]; OR for underground soft rock mining, 2.92 [1.65-5.19]). Table 3 presents the ORs for RA for all mining industry exposures combined as well as for nonmining silica exposures for RA using multiple definitions of disease (eg, OR for corticosteroid-treated RA, 4.12 [95%, 2.49-6.81]; OR for DMARD-treated RA, 3.30 [95% CI, 1.93-5.66]).

**Table 2.** Arthritis Conditions Associated With Silica Exposure

Model	RA plus corticosteroids, excluding non-RA arthritis (model n = 1162)^a^		RA plus DMARD, excluding non-RA arthritis (model n = 1153)^b^		Non-RA arthritis, excluding RA (model n = 1870)	
	No. of events	OR (95% CI)	No. of events	OR (95% CI)	No. of events	OR (95% CI)
Silica exposures unadjusted for other covariates						
Any underground hard rock mining (n = 118)	9	3.41 (1.55-7.48)	5	2.02 (0.76-5.40)	50	1.38 (0.93-2.05)
Underground soft rock, no hard rock mining (n = 62)	8	9.41 (3.83-23.11)	5	6.27 (2.19-17.93)	34	2.92 (1.65-5.19)
Surface mining, no underground (n = 262)	21	3.70 (2.07-6.61)	19	3.57 (1.95-6.53)	108	1.39 (1.05-1.84)
Silica only from nonmining sources (n = 348)	19	2.78 (1.53-5.03)	21	3.27 (1.82-5.87)	167	1.78 (1.39-2.29)
No exposure (n = 1198)	32	1 [Reference]	30	1 [Reference]	438	1 [Reference]
Silica exposure sources adjusted multivariable logistic regression^c^						
No exposure	NA	1 [Reference]	NA	1 [Reference]	NA	1 [Reference]
Any underground hard rock mining	NA	3.21 (1.45-7.10)	NA	1.91 (0.71-5.12)	NA	1.32 (0.89-1.97)
Underground soft rock mining, no hard rock	NA	9.74 (3.89-24.42)	NA	6.52 (2.26-18.80)	NA	3.04 (1.71-5.42)
Surface mining, no underground	NA	3.74 (2.07-6.75)	NA	3.51 (1.90-6.48)	NA	1.43 (1.07-1.90)
Silica only from nonmining sources	NA	3.40 (1.84-6.27)	NA	3.59 (1.97-6.54)	NA	1.92 (1.48-2.48)
Smoke exposure						
Never	NA	1 [Reference]	NA	1 [Reference]	NA	1 [Reference]
Former	NA	1.12 (0.69-1.82)	NA	0.98 (0.59-1.64)	NA	1.40 (1.14-1.71)
Current/recent	NA	1.05 (0.50-2.21)	NA	1.15 (0.56-2.35)	NA	0.92 (0.67-1.28)
Non-Hispanic White^d^	NA	0.77 (0.44-1.34)	NA	0.70 (0.40-1.22)	NA	1.18 (0.92-1.52)
Age per year	NA	1.04 (1.02-1.07)	NA	1.03 (1.002-1.05)	NA	1.02 (1.01-1.03)

Abbreviations: DMARD, disease-modifying antirheumatic drug; RA, rheumatoid arthritis.

^a^ Report of a clinician’s diagnosis of RA and treatment with corticosteroids for joint symptoms.

^b^ Report of clinician’s diagnosis of RA, plus treatment with DMARDs (see the Disease Classification subsection of the Methods section for included medications).

^c^ Adjusted for all variables shown.

^d^ Compared with all other categories.

**Table 3.** Odds Associated With Differing Definitions of RA

Definitions of RA	No. of cases/total No.	OR (95% CI)^a^	
		Any mining exposure	Silica exposure only from nonmining sources
RA plus corticosteroid	89/1162	4.12 (2.49-6.81)	3.39 (1.84-6.25)
RA plus long-term corticosteroids^b^	49/1122	6.08 (3.08-12.02)	4.20 (1.78-9.88)
RA plus DMARDs^c^	80/1153	3.30 (1.93-5.66)	3.56 (1.96-6.49)
RA plus DMARDs or corticosteroids^c^	118/1191	3.46 (2.21-5.40)	3.38 (2.01-5.67)

Abbreviations: DMARD, disease-modifying antirheumatic drug; OR, odds ratio; RA, rheumatoid arthritis (health care clinician’s diagnosis).

^a^ Logistic regression models were adjusted for age, race and ethnicity, and smoking status. Each model excluded respondents who reported arthritis but did not meet the given definition.

^b^ Indicates 3 or more times per week for 3 or more months.

^c^ See the Disease Classification subsection of the Methods section for included medications.

**Figure 2.**
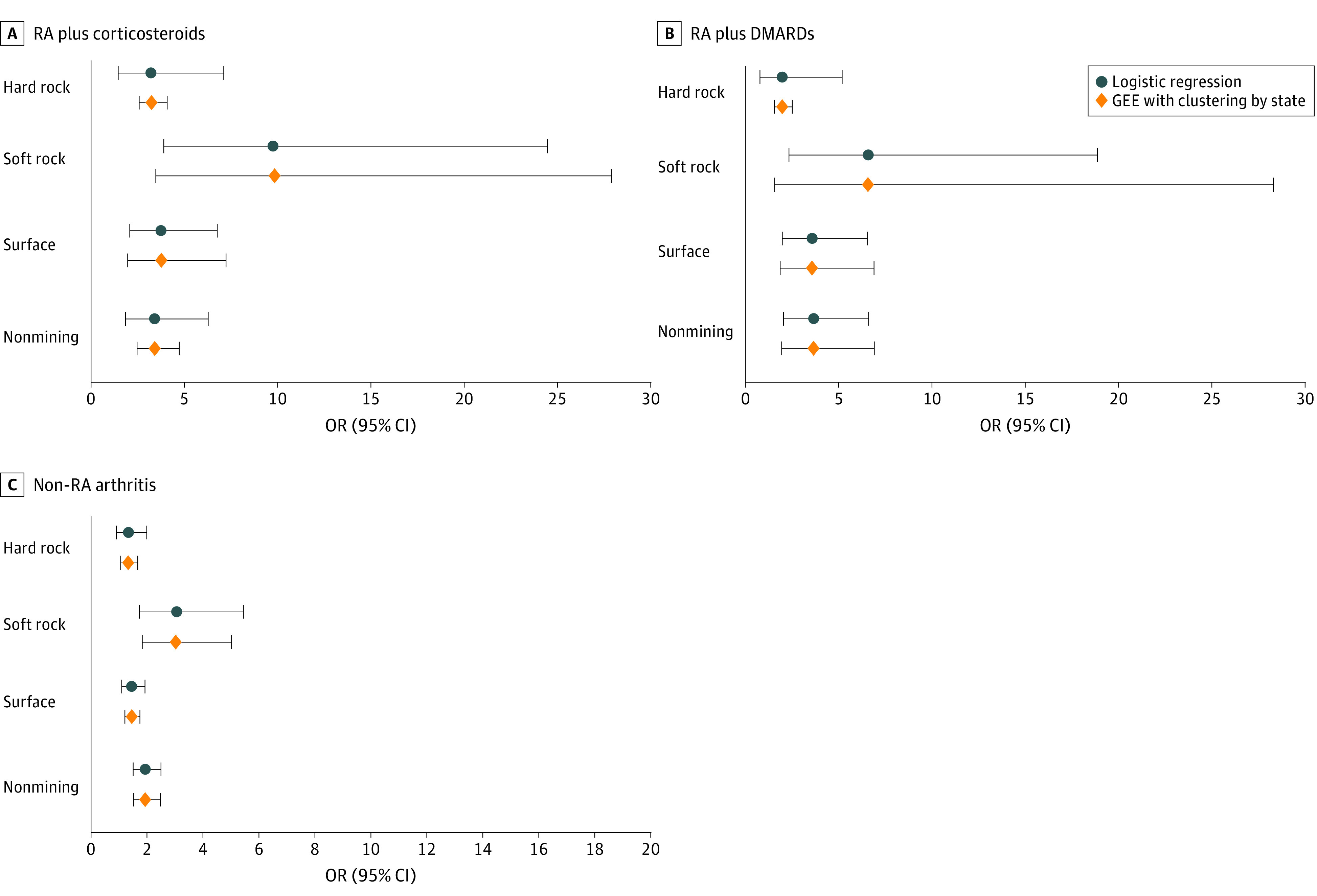
Multivariable Logistic Regression vs Generalized Estimating Equation (GEE) Modeling for Occupational Silica Exposure Exposures include underground hard rock mining, underground soft rock mining, surface mining, and nonmining occupations. A, Corticosteroid treatment–defined rheumatoid arthritis (RA); B, disease-modifying antirheumatic drug (DMARD)–treated RA; and C, arthritis other than RA (non-RA arthritis).

The results from generalized estimating equations modeling accounting for potential clustering by geographic sample (Utah, Colorado, or New Mexico) compared with multivariable models are shown in Figure 2. Although the point estimates for the ORs are very similar from both approaches, for underground hard rock mining the 95% CIs in the generalized estimating equations model for DMARD-defined arthritis and for non-RA arthritis were narrower and excluded 1.00 (Figure 2, B and C). The data are also provided in tabular form (eTable in the Supplement).

## Discussion

In this population-based study across a range of occupations, we identified increased odds of RA associated with underground hard rock mining as well as other underground and above-ground mining industry employment and nonmining work-related silica exposure. Our findings add to the body of evidence suggesting that occupational silica exposure is an important contributor to RA risk among men.^13^

We defined RA based on report of a clinician’s diagnosis and medication therapy but did not have access to confirmatory medical records nor to the results of autoimmune serology. To address this limitation, we used less restrictive and more restrictive RA diagnostic criteria, including a definition of disease requiring treatment with DMARDs. The estimated odds of RA associated with occupational silica exposure were attenuated for hard rock mining in particular using the DMARD definition, but the point estimates of risk were elevated for all of the silica exposure sources when both the less restrictive and more restrictive defining criteria were used, supporting these classifications of disease. The corticosteroid-based definition, although less specific, recognizes that access to rheumatology subspecialists likely to prescribe DMARDs can be limited.^14^ We did not find, however, the expected association of RA with smoking (especially current smoking). Notably, we observed such a smoking association in our previous studies of coal miners in Appalachia,^3,4^ a population with similar proportions of current and prior smokers.

Furthermore, in our study, the point estimate of the odds of RA associated with coal and other underground fossil hydrocarbon mining (predominantly coal) was substantially higher than that observed for underground hard rock mining or other sources of silica. This suggests that in coal mining, silica inhalation may not be the sole cause, but rather that carbonaceous materials may also be involved etiologically in RA risk in that occupation. It is intriguing that black carbon was found to stimulate protein citrullination in vitro to a degree similar to the effect of silica.^15^ Also relevant to this question, recent epidemiologic data suggest that black carbon as a component of fine particulate air pollution may be a risk factor for lung fibrosis in newly diagnosed RA.^16^ The duration of employment in underground coal mining, however, was substantially longer than that in hard rock mining in our study population, which could account for the point estimate of higher odds of disease in the former group and the attenuation of risk for the latter, especially when considering the more restrictive, DMARD-based definition of disease. Our failure to observe the anticipated smoking association in this cohort is not explained by employment duration. Moreover, there appeared to be sufficient prevalence and cumulative intensity of smoking to detect its risk. Nonetheless, the precise components of cigarette smoke that mediate its promotion of RA risk have not been identified. Smoking can be viewed as simply one among a spectrum of environmental inhalants playing a role in RA.^17^ We did not consider the complex question of smoking risk in RA as a secondary study hypothesis.

Other aspects of manual labor, in particular in the mining sector, are likely to carry risk for arthritis other than RA, an association we observed in the present study and in our population-based studies in Appalachia.^3,4^ An analysis of outpatients from a New Mexico clinic with targeted outreach to miners^18^ observed increased relative risks for arthritis among uranium (1.3), metal (1.3), and nonmetal (1.4) miners. This is similar to our estimate for hard rock miners, but less than what we estimated for coal miners and related mining occupations.

### Limitations

This study has some limitations. Although we did not have access to confirmatory employment records, recall bias is unlikely to have influenced respondents’ report of their work histories or their arthritis diagnoses. Misclassification of exposure and misdiagnosis are unlikely to have been systematic and thus would be biased in the direction of failing to reject the null hypothesis. Because our study was geographically centered in the Four Corners region of the US, our findings may not be transferable to other regions and in fact clearly differ from our findings from Appalachia.^3,4^ The narrowed 95% CIs for the ORs for hard rock mining estimated by generalized estimating equations suggest that geographic clustering may have been present for that exposure, but we do not have detailed employment histories identifying specific mine sites at which study participants may have been employed to explore clustering in that regard. Another study limitation is the relatively short duration of employment in underground hard rock mining and the lack of measures available in an industry-specific study such as shifts worked or measurement of dust levels, precluding a refined analysis of hard rock mining duration intensity as reported in a pivotal early study from South African gold mines.^10^ Limiting recruitment to persons 50 years or older may have introduced a selection bias effect in which the participants’ past exposures reflected working conditions that have since changed in a manner reducing risk in a younger cohort. In contrast to age eligibility, because we took a population-based approach that was not based on occupation or diagnosis, there is no reason to assume that selection bias was operative for those factors, for example, that coal miners with arthritis were more likely than any other contacts to agree to be interviewed.

## Conclusions

The findings of this cross-sectional population-based survey study suggest that underground hard rock and coal miners, along with surface mine industry workers, experience 3- to 9-fold increased odds of RA, depending on the definition of disease used. These findings further suggest that clinicians should consider patients with relevant work exposures as being at higher risk for developing RA.

## Notes

Supplement. **eTable.** Multivariable Logistic Regression Results Compared With Models Accounting for State-Level Clustering Using Generalized Estimated Equations **eFigure.** Survey Participant Recruitment and Exclusions **eAppendix.** Survey Instrument: Rheumatoid Arthritis Risk in Underground Hard Rock and Other Mining Industry Workers in Colorado, New Mexico, and Utah
