# Supplementary material for: Assessment of Risk of Rheumatoid Arthritis Among Underground Hard Rock and Other Mining Industry Workers in Colorado, New Mexico, and Utah
Source: JAMA Netw Open. 2022 Oct 17;5(10):e2236738. doi: 10.1001/jamanetworkopen.2022.36738 (PMC9577677; doi:10.1001/jamanetworkopen.2022.36738)
Supplement: Supplement. — eTable. Multivariable Logistic Regression Results Compared With Models Accounting for State-Level Clustering Using Generalized Estimated Equations eFigure. Survey Participant Recruitment and Exclusions eAppendix. Survey Instrument: Rheumatoid Arthritis Risk in Underground Hard Rock and Other Mining Industry Workers in Colorado, New Mexico, and Utah [file jamanetwopen-e2236738-s001.pdf]

Supplementary Online Content

Blanc PD, Trupin L, Yelin EH, Schmajuk G. Assessment of risk of rheumatoid arthritis among underground hard rock and other mining industry workers in Colorado, New Mexico, and Utah. *JAMA Netw Open*. 2022;5(10):e2236738. doi:10.1001/jamanetworkopen.2022.36738

eTable. Multivariable Logistic Regression Results Compared With Models Accounting for State-Level Clustering Using Generalized Estimated Equations
eFigure. Survey Participant Recruitment and Exclusions
eAppendix. Survey Instrument: Rheumatoid Arthritis Risk in Underground Hard Rock and Other Mining Industry Workers in Colorado, New Mexico, and Utah

This supplementary material has been provided by the authors to give readers additional information about their work.

© 2022 Blanc PD et al. *JAMA Network Open*.

eTable. Multivariable Logistic Regression Results Compared With Models Accounting for State-Level Clustering Using Generalized Estimated Equations

	RA+ corticosteroids* (excluding non-RA)		RA+ DMARDs** (excluding non-RA)		Non-RA arthritis (excluding RA*)	
	Adjusted OR (95% CI)					
Silica exposures (referent = no exposure)	Logistic regression	GEE	Logistic regression	GEE	Logistic regression	GEE
Any underground hard rock mining	3.21 (1.45, 7.10)	3.24 (2.57, 4.08)	1.91 (0.71, 5.12)	1.91 (1.49, 2.44)	1.32 (0.89, 1.97)	1.31 (1.04, 1.65)
Any underground soft rock mining	9.74 (3.89, 24.42)	9.82 (3.47, 27.85)	6.52 (2.26, 18.80)	6.50 (1.50, 28.23)	3.04 (1.71, 5.42)	3.01 (1.81, 5.00)
Any surface mining, no underground	3.74 (2.07, 6.75)	3.76 (1.96, 7.22)	3.51 (1.90, 6.48)	3.50 (1.79, 6.82)	1.43 (1.07, 1.90)	1.43 (1.19, 1.72)
Silica only from non-mining sources	3.40 (1.84, 6.27)	3.41 (2.46, 4.72)	3.59 (1.97, 6.54)	3.58 (1.88, 6.84)	1.92 (1.48, 2.48)	1.91 (1.49, 2.45)

© 2022 Blanc PD et al. *JAMA Network Open*.

eFigure. Survey Participant Recruitment and Exclusions

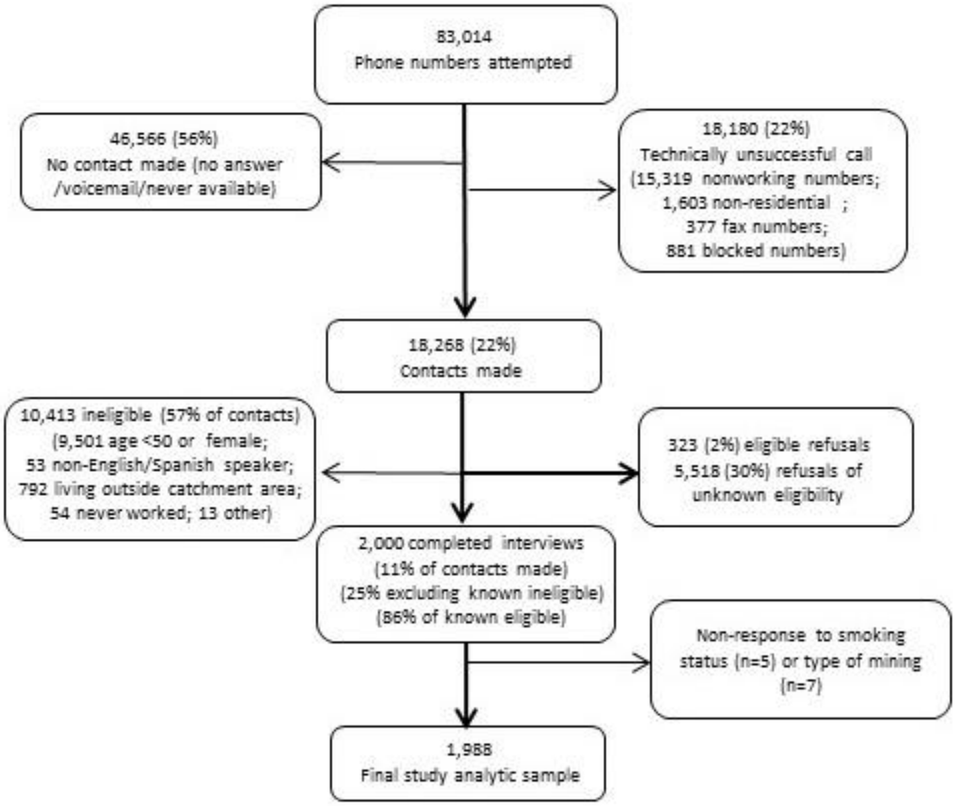

© 2022 Blanc PD et al. *JAMA Network Open*.

eAppendix. Survey Instrument: Rheumatoid Arthritis Risk in Underground Hard Rock and Other Mining Industry Workers in Colorado, New Mexico, and Utah

– Screening Questions –

Hello, my name is _________________, and I am calling from Davis Research, an independent public opinion research organization. We are conducting an important survey for one of the nation's leading schools of medicine about some work and health-related issues. This is a legitimate public opinion survey – we are not selling anything.

S1. We are only conducting this survey in certain states and counties in the U.S. In what state do you live?

COLORADO 1
NEW MEXICO 2
UTAH 3
ALL OTHER STATES...4→**END**
REFUSED .... REF →**END**

S2. And, in what county do you live?

IF COLORADO	IF UTAH	IF NEW MEXICO
DELTA 01	GRAND.................. 01	BERNALILLO.......... 01
DOLORES 02	SAN JUAN 02	CIBOLA 02
GARFIELD 03	UINTAH 03	LOS ALAMOS......... 03
LA PLATA...............04	ALL OTHER 04	MCKINLEY 04
MESA 05	REFUSED ....REF	RIO ARRIBA........... 05
MONTEZUMA..........06		SAN JUAN 06
MONTROSE............07		SANDOVAL 07
RIO BLANCO 08		SANTA FE 08
SAN JUAN..............09		TAOS 09
SAN MIGUEL 10		TORRANCE 10
ALL OTHER 11		VALENCIA 11
REFUSED .... REF		ALL OTHER 12
		REFUSED .... REF

IF S2=OTHER OR REFUSED, ASK:

S2b. What is your zip code?

(CODE TO COUNTY: IF MATCHES CONTINUE. OTHERWISE END)

____
DK/REFUSED .... REF →**END**

S3. **RECORD GENDER:** (INTERVIEWER: I F YOU HAVE ANY DOUBT AS TO RESPONDENT'S GENDER, SAY: Because it is sometimes difficult to determine over the phone, I am asked to confirm whether you are male or female.)

MALE 1
FEMALE 2

© 2022 Blanc PD et al. *JAMA Network Open*.

LANDLINE SAMPLE

IF MAN IS ON THE TELEPHONE, SAY:

S4a. For this interview I need to speak to a male age 50 or older. Does a male age 50 or older live in your household? (IF YES, ASK) Is that you or someone else? (IF SOMEONE ELSE, ASK TO SPEAK TO THAT PERSON) (IF MORE THAN 1 MALE AGE 50 OR OLDER LIVING IN HOUSEHOLD, SAY: I'd like speak with the youngest male age 50 or older who is at home now.)

SPEAKING TO ELIGIBLE MALE……….1 **CONTINUE WITH S5A**
ELIGIBLE MALE COMES TO PHONE……...2 **REPEAT INTRO, THEN CONTINUE WITH S5A**
ELIGIBLE MALE NOT AVAILABLE NOW……3 **CALLBACK**
NO MALES AGE 50+ IN HOUSEHOLD ..4 **END**
REFUSED……………………………REF **END**

IF WOMAN IS ON THE TELEPHONE, SAY:

S4b. For this interview I need to speak to a male age 50 or older. Does a male age 50 or older live in your household? (ASK TO SPEAK TO THAT PERSON) (IF MORE THAN 1 MALE AGE 50 OR OLDER LIVING IN HOUSEHOLD, SAY: I'd like to speak with the youngest male age 50 or older who is at home now.)

ELIGIBLE MALE COMES TO PHONE……. 1 **REPEAT INTRO, THEN CONTINUE WITH S5A**
ELIGIBLE MALE IS NOT AVAILABLE NOW …2 **CALLBACK**
NO MALES AGE 50+ IN HOUSEHOLD 3 **END**
REFUSED……………………………. REF **END**

S5a. What is your age?

________ **IF AGE 50+, GO TO S6A**
IF UNDER AGE 50 .... X → **END**
REFUSED ....REF → **ASK S5B**

IF REFUSED, ASK:

S5b. I don't need to know exactly, but can you tell me if you are under age 50, age 50-54, 55-59, 60-64, 65-69, 70-74 or 75 or older?

UNDER AGE 50 .... 1 →**END**
50-54……………………..2 **CONTINUE**
55-59……………………..3 **CONTINUE**
60-64……………………..4 **CONTINUE**
65-69……………………..5 **CONTINUE**
70-74…………………… .6 **CONTINUE**
75 OR OLDER…………… .7 **CONTINUE**
REFUSED ....REF →**END**

S6a. Did you ever work for pay or profit for one year or longer, including civilian and military duties? (**IF NECESSARY, PROMPT**) This includes civilian or military duties and any job that you worked at for one year or longer, even when self-employed?

YES………………………1 **GO TO S10**
NO ………………………..2 **ASK S6B**
NO ANSWER/REFUSED …..REF **ASK S6B**

IF NO OR REFUSED, ASK:

S6b. Did you ever work without salary or pay on a farm or family business for one year or more?

YES………………………1 **GO TO S10**
NO ………………………..2 **END**
NO ANSWER/REFUSED …..REF **END**

© 2022 Blanc PD et al. *JAMA Network Open*.

CELL PHONE SAMPLE

- **IF MALE FROM S3, GO TO S8A; IF FEMALE FROM S3, END**

S8a.	What is your age?	_______ **IF AGE 50+, GO TO S9A**<br>IF UNDER AGE 50…………X →**END**<br>REFUSED………………… REF →**ASK S8b**

IF REFUSED, ASK:

S8b.	I don't need to know exactly, but can you tell me if you are under age 50, age 50-54, 55-59, 60-64, 65-69, 70-74 or 75 or older?	UNDER AGE 50……………. 1 →**END**<br>50-54……………………..2 **CONTINUE**<br>55-59……………………..3 **CONTINUE**<br>60-64……………………..4 **CONTINUE**<br>65-69……………………..5 **CONTINUE**<br>70-74……………………..6 **CONTINUE**<br>75 OR OLDER…………… .7 **CONTINUE**<br>REFUSED………………… REF →**END**

S9a.	Did you ever work for pay or profit for one year or longer, including civilian and military duties? (**IF NECESSARY, PROMPT**) This includes civilian or military duties and any job that you worked at for one year or longer, even when self-employed?	YES……………………….1 **GO TO S10**<br>NO ………………………..2 **ASK Q9B**<br>NO ANSWER/REFUSED …..REF **ASK Q9B**

IF NO OR REFUSED, ASK:

S9b.	Did you ever work without salary or pay on a farm or family business for one year or more?	YES……………………….1 **GO TO S10**<br>NO ………………………..2 **END**<br>NO ANSWER/REFUSED …..REF **END**

IF ELIGIBLE FROM S6 OR S9, SAY

S10.	You are eligible to participate in an important study about the impact of work on health. Upon completing the survey, we will send you a $5 gift card to Amazon or Starbucks or we can mail you a check . . . . It will not take long, only about 15-20 minutes for most people . . . . It is completely voluntary and you can stop at any point. . . . The study's principal investigator is Dr. Paul Blanc at the University of California, School of Medicine. Would you like to participate?	YES, PROCEED ………………… 1 → **CONTINUE**<br>YES, BUT CALL BACK LATER ….. 2 → **ARRANGE CALLBACK**<br>UNSURE/HAS QUESTIONS …….. 3 → **READ TEXT BELOW**<br>NO, REFUSED …………………REF →**END**

(**IF UNSURE OR HAS QUESTIONS, SAY**) If you have any questions, we can give you the telephone number of Dr. Blanc's office or for the Office of Research Affairs at the University. (**IF REQUESTED**) Dr. Blanc's research office telephone number is 415-476-7377. The University Office of Research Affairs telephone number is 415-476-1814.

IF CELL PHONE LISTING, ASK

S11.	For your safety, are you currently driving a motor vehicle, operating heavy equipment or in a place that is unsafe to do the survey?	YES,……………………..……1→ **ARRANGE CALLBACK**<br>NO………………………..2→ **CONTINUE**

IF CALLBACK FROM S10 OR S11, ASK:

S12a.	When would be a good time for us to call back?	**RECORD DAY AND TIME OF CALLBACK**
S12b.	So our interviewer can ask for you to speak to (that person) (you) by name, what is (his) (your) first name?	**RECORD FIRST NAME FOR CALLBACK**

Main Survey

Before we begin, I need to tell you that my supervisor sometimes monitors these interviews to ensure quality and courtesy.

The first questions concern work and employment.

1. Are you currently employed for pay or profit either full or part time?
   - YES ....1 (ASK Q1A)
   - NO............................2 (SKIP TO Q2)
   - NO ANSWER/REFUSED...DK (SKIP TO Q2)

IF Q1=YES, ASK:

1a. Do you currently work in underground hardrock mining?
   - YES.........................1 (SKIP TO Q3)
   - NO ....2 (ASK Q2)
   - NO ANSWER/REFUSED . DK (ASK Q2)

IF Q1=NO OR DK OR Q1A=NO OR DK, ASK:

2. Did you ever work in underground hardrock mining?
   - YES ....1 (ASK Q3)
   - NO...........................2 (SKIP TO Q11)
   - NO ANSWER/REFUSED...DK (SKIP TO Q11)

3. For how many years altogether did you do this work? ____ (years) DK/REF. . . .DK

4. Which of the following underground hardrock mining have you done: **(READ ITEMS IN RANDOM ORDER, ASKING)** Have you done mining for **(ITEM)** ?

	YES	NO	DK/REF
a. Silver	1	2	DK
b. Gold	1	2	DK
c. Copper	1	2	DK
d. Uranium or Zircon	1	2	DK
e. Molybdenum (mah-LIB-duh-num)	1	2	DK

IF Q4A-E ALL =NO OR DK, ASK:

f. Another mineral (other than coal)? **(IF NECESSARY)** We will be asking you a little later about coal	1	2	DK

IF YES: What was that? ____________________ DK/REF. . . .DK

5. Did you work for a period as a hardrock mining apprentice or nipper?
   - YES............................1 (ASK Q5a)
   - NO ....2 (SKIP TO Q6)
   - NO ANSWER/REF..... DK (SKIP TO Q6)

IF Q5=YES, ASK:

5a. For how many years did you do this work?
   - ________ YEARS
   - NO ANSWER/REFUSED .... DK

6. Did you work as a stope development miner?

YES .... 1 (ASK Q6a)
NO.................... .....2 (SKIP TO Q7)
NO ANSWER/REF.....DK (SKIP TO Q7)

IF Q6=YES, ASK:

6a. For how many years did you do this work?

________ YEARS
NO ANSWER/REF .... DK

6b. Did you do your own mucking?

YES.................1
NO....... 2
NO ANSWER/REF..DK

7. Did your underground hardrock work include any blasting jobs?

YES .... 1 (ASK Q7a)
NO.................... .....2 (SKIP TO Q8)
NO ANSWER/REF.....DK (SKIP TO Q8)

IF Q7=YES, ASK:

7b. Specifically, did you work as a (ITEM)? **(READ IN ORDER UNTIL 1ST "YES", THEN SKIP TO Q7C)**

		YES	NO	DK/REF
(1).	Blaster or blaster helper	1	2	DK
(7).	High-raise blaster	1	2	DK
(3).	Powderman or shotman	1	2	DK
(4).	Bench blaster	1	2	DK
(5).	Chute blaster	1	2	DK
(6).	Hang-up blaster	1	2	DK
(2).	Shooter or shooter helper	1	2	DK

IF Q7b (1)-(7) ALL = NO OR DK, ASK:

viii. Did you work in any other blasting jobs? ....1 ....2 .. ...DK

IF YES: What was that? ____________________ DK/REF. . . .DK

7c. For how many years did your hardrock mining career include blasting jobs?

________ YEARS
NO ANSWER/REFUSED DK

8. Did your underground hardrock work include any drilling or cutting jobs?

YES .... 1 (ASK Q8A)
NO.................... ..... 2 (SKIP TO Q9)
NO ANSWER/REF.....DK (SKIP TO Q9)

© 2022 Blanc PD et al. *JAMA Network Open*.

IF **Q8**=YES, ASK:

8a. Specifically, did you work as a **(ITEM)**? **(READ IN ORDER UNTIL 1ST "YES", THEN SKIP TO Q8d, OTHERWISE ASK Q8B)**

		YES	NO	DK/REF
(1).	Drift driller or drift miner	1	2	DK
(3).	Long-hole driller	1	2	DK
(4).	Block holer	1	2	DK
(2).	Blast hole driller	1	2	DK
(5).	Rock bolter	1	2	DK
(6).	Diamond driller or drill runner	1	2	DK
(7).	In the hole driller	1	2	DK
(8).	Stoper driller	1	2	DK

8b. Did you work as an operator of a **(ITEM)**? **(READ IN ORDER UNTIL 1ST "YES", THEN SKIP TO Q8d)**

		YES	NO	DK/REF
(1).	Bolter	1	2	DK
(2).	Core-drill	1	2	DK
(3).	Raise driller or boring machine	1	2	DK
(4).	Ring drill	1	2	DK
(5).	Road header	1	2	DK
(6)	In-hole drill	1	2	DK
(7).	Jack leg drill	1	2	DK
(8).	Jumbo drill	1	2	DK
(9).	Wagon drill	1	2	DK
(10).	Undercutter	1	2	DK

IF **Q**8a(1)-(8) **AND** Q8b **(1)- (10) ALL = NO OR DK, ASK:**

8c. Did you work in any other driller or drill machine operator job?............................1 ..... …2 …DK

IF YES: What was that? ____________________ DK/REF. . . .DK

8d. For how many years did your hard rock mining career include drilling jobs?

________ YEARS
NO ANSWER/REFUSED DK

8e. For how much of the time did this drilling work use water to control dust – almost none of the time, sometimes, most of the time, or almost always?

ALMOST NONE OF THE TIME…………..……………1
SOMETIMES..………………….. 2
MOST OF THE TIME …………. 3
ALMOST ALWAYS……………… .4
NO ANSWER/REF……………DK

9. Did your underground hardrock work include any mined material handling, loading or hauling jobs?

YES .... 1 (ASK Q9a)
NO.................. …..2 (SKIP TO Q11)
NO ANSWER/REF ... DK (SKIP TO Q11)

© 2022 Blanc PD et al. *JAMA Network Open*.

IF **Q9=YES, ASK:**

9a. Specifically, did you work as a **(ITEM)**? **(READ IN ORDER UNTIL 1ST "YES", THEN SKIP TO Q9d, OTHERWISE ASK Q9b)**

		YES	NO	DK/REF
(3).	Diesel loader	1	2	DK
(1).	Cager	1	2	DK
(2).	Downhole loader	1	2	DK

9b. Did you work as an operator of a **(ITEM)**? **(READ IN ORDER UNTIL 1ST "YES", THEN SKIP TO Q9d)**

		YES	NO	DK/REF
(7).	Loading machine	1	2	DK
(1).	Grader	1	2	DK
(2).	Crusher	1	2	DK
(3).	Grizzly	1	2	DK
(4).	Dozer or jammer	1	2	DK
(5).	Front-end loader	1	2	DK
(6).	Load-haul dump	1	2	DK
(8).	Slusher	1	2	DK
(9).	Mucker or mucker machine	1	2	DK
(10).	Production loader	1	2	DK
(11).	Scoop or scoop tram	1	2	DK
(12).	Locomotive	1	2	DK
(13).	Haul truck	1	2	DK

IF Q9a (1)-(4) AND Q9b (1)- (13) ALL = NO OR DK, ASK:

9c. Did you work in any other loading, handling or hauling jobs? ....1 ....2 . . . DK

IF YES: What was that? ____________________ DK/REF. . . .DK

9d. For how many years did your hardrock career include mined material handling, loading or hauling?

________ YEARS
NO ANSWER/REFUSED DK

NOTE: Q10 AND Q10A HAVE BEEN DELETED

11. Did you work in open-pit hardrock mining?

YES............................ 1 (ASK Q11a)
NO ....2 (SKIP TO Q12)
NO ANSWER/REF .... DK (SKIP TO Q12)

IF Q11=YES, ASK:

11a. Considering all open pit hardrock mining, for how many years in total did you do this work?

________ YEARS
NO ANSWER/REF............. DK

12. Did you work in ore processing or metal refining?

YES ....1 (ASK Q12a)
NO ....2 (SKIP TO Q13)
NO ANSWER/REF... DK (SKIP TO Q13)

IF Q12=YES, ASK:

12a. Considering all hardrock mining, for how many years in total did you do this processing or refining work?

________ YEARS
NO ANSWER/REF............. DK

13. Have you ever worked in a quarry or sand pit?

YES .... 1 (ASK Q13a and b)
NO......................2 (SKIP TO Q14)
NO ANSWER/REF.....DK (SKIP TO Q14)

© 2022 Blanc PD et al. *JAMA Network Open*.

IF Q13=YES, ASK:

13a. Did this include granite, quartz, sandstone or sand?
YES 1
NO 2
DON'T KNOW/REFUSED .... DK

13b. Considering all quarrying or sand pit work, for how many years did you do this work?
________ YEARS
NO ANSWER/REF ....DK

14. Have you ever worked in coal mining?
YES..............................1 (ASK Q15)
NO.......................…..2 (SKIP TO Q17)
NO ANSWER/REF .... DK (SKIP TO Q17)

IF Q14=YES, ASK:

15. Did this include underground coal mining?
YES.......................... 1 (ASK Q15a)
NO.................. …..2 (SKIP TO Q16)
NO ANSWER/REF ... DK (SKIP TO Q16)

IF Q15=YES, ASK:

15a. Were you ever a coal mine roof bolter?
YES 1
NO 2
NO ANSWER/REFUSED................................. DK

15b. Did you ever work in underground coal mine construction or development?
YES ....1 (ASK Q15bI)
NO .... 2 (GO TO Q15C)
NO ANSWER/REFUSED........... DK (GO TO Q15C)

IF Q15b = YES, ASK:

15b(1). Did you cut through rock, for example in coal mine slope or shaft construction?
YES...1
NO 2
NO ANSWER/REFUSED ....DK

15c. Considering all underground coal mining, for how many years in total did you do this work?
________ YEARS
NO ANSWER/REFUSED .... DK

15d. What percentage of these years did you work at the coal face?
________ PERCENT (O%-100%)
NO ANSWER/REFUSED................................. DK

© 2022 Blanc PD et al. *JAMA Network Open*.

16. Did your coal mining work ever include surface or strip mining?

YES....................................1 (GO TO Q16a)
NO ....2 (GO TO Q17)
NO ANSWER/REFUSED ....DK (GO TO Q17)

IF Q16=YES, ASK:

16a. In this work did you operate a bulldozer, dragline, or scraper?

YES..1
NO 2
NO ANSWER/REFUSED ....DK

16b Were you a high wall or auger operator or helper?

YES..1
NO 2
NO ANSWER/REFUSED ....DK

16c. Considering all surface or strip coal mining, for how many years in total did you do this work?

__________ YEARS
NO ANSWER/REFUSED……………DK

17. Has a medical doctor ever told you that you have pneumoconiosis, silicosis or black lung?

YES……………………..1
NO………………………2
NO ANSWER/REF………DK

18. Thinking about all of the types of work other than hardrock or coal mining that you have done for <u>one year or more</u> during your career, did any non-mining job involve regular exposure to breathing dusty air?

YES. ....1 (GO TO Q18a)
NO ....2 (GO TO Q19)
NO ANSWER/REF ...DK (GO TO Q19)

IF Q18=YES, ASK:

18a. For how many years did any non-mining job regularly expose you to breathing dusty air?

________ YEARS
NO ANSWER/REFUSED.........DK

18b. Did you ever have regular contact on a non-mining job with any of the following? (**READ ITEMS ONE AT A TIME IN RANDOM ORDER**)

		YES	NO	DK/REF
(1).	Silica, sand, or concrete dust	1	2	DK
(2).	Sandblasting	1	2	DK
(3).	Explosives or blasting fumes	1	2	DK
(4).	Foundry work	1	2	DK
(5).	Concrete finishing, cutting, or drilling	1	2	DK
(6).	Masonry work or tip-pointing	1	2	DK
(7).	Metal grinding or polishing	1	2	DK
(8).	Construction dust	1	2	DK
(9).	Soil or agricultural dust	1	2	DK

19. Thinking about all of the types of work that you have done for five years or longer during your career… Did this include (**ITEM**) on an almost daily basis? (**READ ITEMS ONE AT A TIME IN RANDOM ORDER**)

	YES	NO	DK/REF
(1) lifting, carrying, bending, stooping, pulling or pushing	1	2	DK
(2) bending, kneeling, squatting, treadle work or extensive standing or walking	1	2	DK
(3) using vibrating, pneumatic (noo-mat-ik) or hand tools	1	2	DK
(4) working with your arms overhead or reaching, or your neck twisted or bent	1	2	DK
(5) gripping, bending or pinching work with your hands	1	2	DK

Now, some questions about your health.

20. In general, would you say your health is excellent, very good, good, fair, or poor?

- EXCELLENT 1
- VERY GOOD 2
- GOOD 3
- FAIR 4
- POOR 5
- NO ANSWER/DON'T KNOW .... DK

21. Are you limited in any way in any activities because of a long-term physical condition? (**DO NOT COUNT RETIREMENT AS A LONG-TERM HEALTH PROBLEM**)

- YES 1
- NO 2
- DON'T KNOW/REFUSED .... DK

22. Has a doctor, nurse, or other health professional EVER told you that you have arthritis?

- YES .... 1 (ASK Q22A-E)
- NO .... 2 (GO TO Q23)
- NO ANS/REF .... DK (GO TO Q23)

(**IF Q22 = YES, ASK:**)

	YES	NO	DK/REF
a. Was this osteoarthritis (os-tee-oh-arth-right-us) or degenerative arthritis?	1	2	DK
b. Was this rheumatoid (rue-ma-toyed) arthritis?	1	2	DK
c. Was this arthritis due to psoriasis (sor-eye-ah-sis)?	1	2	DK
d. Was this arthritis due to gout?	1	2	DK

e. At what age did your arthritis first start? AGE: ____________

NO ANSWER/REFUSED .. DK

23. Has a doctor, nurse or other health professional EVER told you that you have any of the following medical conditions? (**READ IN RANDOM ORDER**)

	YES	NO	DK/REF
a. Lupus (LOOP-us) or SLE?	1	2	DK
b. Scleroderma (sklare-oh-DERM-ah)?	1	2	DK

24. Does pain, swelling, stiffness or aching regularly affect your (**READ IN RANDOM ORDER**)?

	YES	NO	DK/REF
a. Hands or wrists?	1	2	DK
b. Hips?	1	2	DK
c. Knees?	1	2	DK
d. Back?	1	2	DK
e. Neck?	1	2	DK
f. Ankles or feet?	1	2	DK

(**IF YES TO ANY Q25A-F, ASK:**)

25. For the pain, swelling or stiffness you just identified, have you ever been given . . .?

	YES	NO	DK/REF

© 2022 Blanc PD et al. *JAMA Network Open*.

a. Prednisone (PRED-nuh-zone) or steroid pills? .... 1 ....2..... DK

IF Q25=YES, ASK:

a(1).	Was this at least 3 times a week for 3 months or more?	YES .... 1<br>NO... 2<br>NO ANSWER/REFUSED .... DK

b. Steroid injections into your muscles or joints? .... 1 ....2..... DK

(IF Q22B-C OR Q23A-B OR (Q22= YES AND Q25A OR Q25B=YES), ASK Q26A-F AND Q27A:)

26. Have you ever been given any of the following medicines…**(READ ALL ITEMS IN RANDOM ORDER)**?

	YES	NO	DK/REF
a. Methotrexate (meth-oh-TREX-ate), Rheumatrex (ROOM-ah-trex), Trexall (TREX-all), Otrexup (oh-TREX-up), or Rasuvo (rah-SOOV-oh)..............	1	2	DK
b. Sulfasalazine (sulf-ah-SAL-uh-zeen) or Azulfidine (ay-ZUL-fih-deen)....	1	2	DK
c. Plaquenil (PLA-kwen-ill) or Hydroxychloroquine (hi-drox-ee-KLOR-oh-kwin)	1	2	DK
d. Azathioprine (AY-zah-THIGH-oh-prin), Imuran (IM-your-an), or Azasan (AY-zah-sahn)	1	2	DK
e. Arava (uh-RAVE-uh) or Leflunomide (leh-FLOON-oh-mide)	1	2	DK
f. Xeljanz (ZEL-janz) or Tofacitinib (TOE-fah-SIT-in-ib)	1	2	DK
g. Rinvoq (RIN-vok) or Upadacitinib (up-ada-CIT-in-ib)	1	2	DK
h. Olumiant (OL-um-eh-ant)...	1	2	DK

© 2022 Blanc PD et al. *JAMA Network Open*.

27a. (**IF Q25b=YES, ADD:** Other than the steroid injections you mentioned earlier) have you ever been treated with any injectable medications for arthritis or autoimmune disease?................................1 ....2...... DK

(IF Q27a = YES, ASK:)

27b. Has this included . . . **(READ IN ORDER UNTIL 1ST"YES", THEN SKIP TO Q28)**?

	YES	NO	DK/REF
(1). Enbrel (EN-brel) or Etanercept (ee-TAN-er-cept)	1	2	DK
(9). Cosentyx (koh-SEN-tix) or Secukinumab (sek-you-KIN-you-mab)	1	2	DK
(3). Remicade (REM-ih-kaide), Infliximab (in-FLIX-ih-mab), or Inflectra (In – FLECK-trah)	1	2	DK
(2). Humira (hugh-MEER-uh) or adalimumab (ah-duh-LIM-you-mab)	1	2	DK
(4). Rituxan (rih-TUX-an), Rituximab (rih-TUX-ih-mab), or Truxima (truck-ZEEM-ah)	1	2	DK
(5). Simponi (sim-POHN-ee) or Golimumab (go-LIM-you-mab)	1	2	DK
(6). Cimzia (SIM-zee-ah) or Certulizumab (sert-uh-LIZ-oo-mab)	1	2	DK
(7). Actemra (ack-TEM-rah) or Tocilizumab (toe-see-LIZ-oo-mab)	1	2	DK
(8). Orencia (oh-REN-see-yah) or Abatacept (ab-AT-ah-sept)	1	2	DK
(10). Stelara (steh-LAH-rah) or Ustekinumab (ooh-steh-KIN-you-mab)	1	2	DK

28. Have you smoked at least 100 cigarettes in your entire life?

YES..1
NO 2
NO ANSWER/REFUSED ....DK

IF Q.28 = YES, ASK:

a.	About how old were you when you first started smoking cigarettes?	_____________ YEARS OLD<br>NO ANSWER/REFUSED......................DK
b.	Do you now smoke cigarettes every day, some days, or not at all?	EVERY DAY ....1<br>SOME DAYS ....2<br>NOT AT ALL..3<br>NO ANSWER/REFUSED......................DK
c.	On the average, over the years you smoked, about how many cigarettes did you smoke a day?	_____________ CIGARETTES<br>NO ANSWER/REFUSED......................DK
d.	Not counting years you may have quit, for how many years altogether (have you smoked) (did you smoke) cigarettes?	_____________ YEARS<br>NO ANSWER/REFUSED......................DK

© 2022 Blanc PD et al. *JAMA Network Open*.

And finally, some questions about yourself for classification purposes.

29. What is the highest level of school that you have completed or the highest degree that you have received? **(READ LIST ONLY IF NECESSARY)**

4TH GRADE OR LESS	1
5TH-8TH GRADE	2
9TH-12TH GRADE (NO HIGH SCHOOL DEGREE)	3
HIGH SCHOOL GRADUATE	4
SOME COLLEGE / NO DEGREE	5
ASSOC. DEGREE / TRADE OR VOCATIONAL SCHOOL	6
COLLEGE GRADUATE OR HIGHER	7
NO ANSWER/REFUSED	DK

30. Are you married, separated or divorced, widowed, never been married, or are you a member of an unmarried couple?

MARRIED	1
SEPARATED/DIVORCED	2
WIDOWED	3
NEVER BEEN MARRIED	4
UNMARRIED COUPLE	5
NO ANSWER/REFUSED	DK

31. How many people, including yourself, live in your household?

____________________	
NO ANSWER/REFUSED	DK

32. For classification purposes, are you Latino or of Hispanic origin or descent?

YES	1
NO	2
NO ANSWER/REFUSED	DK

33. What is your racial background? Are you white, black or African-American, Asian or Pacific Islander, Native American or are you a member of another race? **(ANSWER CAN BE A MULTIPLE)**

WHITE/CAUCASIAN	1
BLACK/AFRICAN-AMERICAN	2
ASIAN/PACIFIC ISLANDER	3
LATINO/HISPANIC (VOLUNTEERED)	4
NATIVE AMERICAN	6
OTHER (SPECIFY) ______________	5
NO ANSWER/REFUSED	DK

34. About how tall are you without shoes?

________________ FEET	
________________ INCHES	
NO ANSWER/REFUSED	DK

35. About how much do you weigh without shoes?

________________ LBS.	
NO ANSWER/REFUSED	DK

36. We don't need to know exactly, but just roughly could you tell me if your annual household income from all sources before taxes in 2020 was less than $20,000, $20,000 through $40,000, $40,000 through $60,000, $60,000 through $80,000, $80,000 through $100,000, or $100,000 or more?

LESS THAN $20,000	1
$20,000 – $39,999	2
$40,000 – $59,999	3
$60,000 – $79,999	4
$80,000 -- $99,999	5
$100,000 OR MORE	6
NO ANSWER/REFUSED	DK

© 2022 Blanc PD et al. *JAMA Network Open*.

37. Is the telephone that I dialed to reach you a landline phone or a cell phone?

LANDLINE PHONE 1
CELL PHONE 2
OTHER.. 3
DON'T KNOW/REFUSED.................... DK

IF Q37=LANDLINE, OTHER OR DK, ASK:

37a.	Do you make and receive personal calls from a cell phone?	YES.. 1<br>NO .... 2<br>DON'T KNOW/REFUSED .... DK

IF Q37=CELL PHONE, ASK:

37b.	Is there a telephone inside your home that you use to make and receive personal calls that is not a cell phone?	YES...................................... 1<br>NO .... 2<br>DON'T KNOW/REFUSED .... DK

IF Q37a OR Q37b =YES, ASK:

37c.	Of all the personal telephone calls that you receive, do you get most of them on a landline phone or on a cell phone?	MOST ON LANDLINE PHONE .... 1<br>MOST ON CELL PHONE .... 2<br>ABOUT EQUAL (VOLUNTEERED)3<br>DON'T KNOW/REFUSED .... DK

38. These are all the questions I have. To thank you for your participation, we would like to send you a $5 gift card from Amazon or Starbucks. What email address should we send that to?

PROVIDES EMAIL ADDRESS…………1 (GO TO Q38A)
NO EMAIL/REFUSES EMAIL…..…………2 (GO TO Q38C)
REFUSES INCENTIVE………REF (GO TO END)

IF Q38=1 (PROVIDES EMAIL), ASK:

38a.	**ENTER EMAIL ADDRESS (READ BACK SLOWLY TO CONFIRM)**	EMAIL:_________________________
38b.	Which would you prefer, an Amazon or a Starbucks gift card?	AMAZON…………………..1 (GO TO END)<br>STARBUCKS………………2 (GO TO END)<br>REFUSES INCENTIVE…REF (GO TO END)

IF Q38= 2 (DOES NOT PROVIDE EMAIL), ASK:

38c.	Then we can mail you your $5 gift card or a check. Which would you prefer – a $5 Amazon gift card, a $5 Starbucks gift card or a check?	AMAZON GIFT CARD ....1<br>STARBUCKS GIFT CARD..........2<br>CHECK.................................3<br>REFUSES INCENTIVE... REF (GO TO END)

38d. What is your name and mailing address? **(ENTER ADDRESS, READ BACK SLOWLY TO CONFIRM)**

NAME: ______________________________________

ADDRESS: ______________________________________

CITY: ______________________________________ ***(CATI DISPLAYS STATE)***

ZIP CODE: ______________________________________ ***(ACCEPT ELIGIBLE ZIP CODES)***

Thank you very much for participating in this important survey.

© 2022 Blanc PD et al. *JAMA Network Open*.
